# Prevalence and pattern of refractive error and visual impairment among schoolchildren: the Lhasa childhood eye study

**DOI:** 10.1186/s12886-021-02134-8

**Published:** 2021-10-12

**Authors:** Jiantao Cui, Jing Fu, Lei Li, Weiwei Chen, Zhaojun Meng, Han Su, Yao Yao, Wei Dai

**Affiliations:** 1 Beijing Tongren Eye Center, Beijing Tongren Hospital, Capital Medical University, Beijing Key Laboratory of Ophthalmology & Visual Sciences, No.1, Dong Jiao Min Xiang Street, Dongcheng District, Beijing, 100730 China; 2 Beijing Institute of Ophthalmology, Beijing, China

**Keywords:** Refractive error, Visual impairment, School children, Myopia, Lhasa

## Abstract

**Background:**

Early and effective ocular screening may help to eliminate treatable eye disorders. The Lhasa Childhood Eye Study (LCES) revealed the particular prevalence of refractive error and visual impairment in grade one schoolchildren (starting age of 6 years old) in Lhasa.

**Methods:**

This is a cross-sectional part of school-based cohort study. One thousand nine hundred forty-three children were enrolled (median age, 6.78 years, range, 5.89 to 10.32). Each child underwent general and ocular examinations, including logarithm of the minimum angle of resolution (logMAR) visual acuity, cycloplegic autorefraction, and slit-lamp biomicroscopy evaluation. Multivariate and correlation analyses were performed to evaluate the association between refractive error with gender and ethnics.

**Results:**

The prevalence of visual impairment (logMAR visual acuity ≥0.3 in the better-seeing eye) of uncorrected, presenting and best-corrected visual acuity (BCVA) was 12.2, 11.7 and 2.7%, respectively. Refractive error presented in 177 (78.0%) out of 227 children with bilateral visual impairment. Myopia (spherical equivalent refractor [SER] ≤ − 0.50 diopter [D] in either eye) was present in 4.7% children when measured after cycloplegic autorefraction. Hyperopia (SER ≥ + 2.00 D) affected 12.1% children. Hyperopia was significantly associated with female gender (*P*<0.001). Astigmatism (cylinder value ≤ − 0.75 D) was present in 44.8% children. In multivariate regression and correlation analysis, SER had no significant difference between ethnic groups.

**Conclusion:**

The Lhasa Childhood Eye Study is the first school-based cohort study to reveal the prevalence and pattern of refractive error and visual impairment in Lhasa. Effective strategies such as corrective spectacles should be considered to alleviate treatable visual impairment.

## Background

Vision impairment caused by refractive error, strabismus, amblyopia and other visual disabilities in children is relatively increased in recent years [1]. Pediatric vision screening is important in the early detection of children who have reduced visual acuity or risk factors that endanger eye health. Effective vision screening enables early treatment of preventable and curable ocular diseases. World Health Organization, American Academy of Ophthalmology and other ophthalmic associations recommend early and regular vision screening throughout childhood [2].

Among the numerous visual impairment disorders, uncorrected refractive error leads to a key problem of heavy burden on learning, employment, and quality of life [3]. Thus, more attention is being paid to prevent and alleviate uncorrected refractive error especially in school children. More important, other ocular conditions, such as amblyopia and strabismus, will obtain favorable therapeutic effect being treated at early age. Worldwide, the prevalence of visual impairment among school-age children differs due to country, region, ethnicity, social development level, medical resources, etc. [1, 4] Evidence shown that refractive error is a concerning issue in China. And different prevalence of refractive error was reported in different regions throughout China [5, 6]. Tibetan adolescents were reported have a lower prevalence of refractive errors compared to China inland without cycloplegia [7]. However, there is no school-based cohort study about school children refraction status follow up from first grade in Tibetan have been published. Reliable evidence is necessary for local public health strategy conducting.

Tibet is characterized by its high-altitude (an average of >3500 m), low air pressure and oxygen concentration, intense solar infrared and ultra-violet radiation, which are obviously different from China inland. Other distinguishing features of Tibetan include a relative lower education level than China inland, lower population mobility and easy to follow up. The differences of regional environment and economics are reported to have influence on human body development and disease composition [8]. Whether the above unique differences affect pediatric eye diseases is not known until recently.

The ethnicity of elementary school children in Lhasa, the provincial capital of Tibet, mainly consists of Tibetan (90. 7%), Han (8.2%) and other minority nationality (1.1%). Lhasa Childhood Eye Study is a school-based cohort study mainly designed to longitudinally observe the occurrence and development of different ocular diseases especially refractive error and visual impairment in school-aged children in Lhasa [9].

## Methods

### Informed consent and ethics approval

This research was reviewed by an independent ethical review board and conforms with the principles and applicable guidelines for the protection of human subjects in biomedical research. The protocols in this study were approved by the Institutional Review Board of Beijing Tongren Hospital, Capital Medical University (TRECKY2019–146), and adhere to the principles of the Declaration of Helsinki. This present study is part of Lhasa Childhood Eye Study, which has registered on Chinese Clinical Trial Registry (http://www.chictr.org.cn, ChiCTR1900026693). Lhasa Childhood Eye Study is mainly designed to investigate the prevalence and associated factors longitudinally of several ocular disorders in school-age children in Lhasa for continuous 5 years. Written informed consent was obtained from a parent or legal guardian of each child prior to the examinations.

### Study setting and population

Stratified random cluster sampling was employed in selecting schools and eligible children for participation. Elementary schools were stratified into three levels based on the evaluation of local government. A total of 1943 grade one students from 14 randomly selected classes represented 7 of the 27 elementary schools available joining the Lhasa Childhood Eye Study.

An experienced clinical team comprising 2 optometrists and 3 ophthalmologists from Beijing Tongren Hospital performed all examination procedures during the period from October to November 2019. All the examinations were performed in the health examination station of Lhasa Maternal and Child Health Care Center.

As Tibetan children were reported to have a lower prevalence of refractive errors than inland, we use a cumulative incidence of 40% to calculate the sample size [7, 9]. Assuming a design effect of 2.0, a tolerated error of 0.1 times the myopia incidence, and a loss of follow-up of 20%, a total of 1382 grade one students would be needed. Further detail of the method is available elsewhere [9].

### Ocular examinations

Demographic data (including ethnicity, age and gender) was collected before examination. All participants underwent standardized ocular examinations included distant visual acuity and identification of visual impairment. Quality controls were implemented throughout the entire study. The detailed standard operation procedures was reported elsewhere [9].

### Distant visual acuity

The ophthalmic examinations included measures of visual acuity and refraction. Distant visual acuity of both eyes was measured at 3 m using a Lea Symbols ETDRS 3-m Set charts (250,300, Goodlite, IL, USA). Children were examined monocularly, right eye followed by left eye. A letter-by-letter logMAR visual acuity score was documented and calculated accordingly. For children wearing glasses, both presenting visual acuity (with glasses) and uncorrected visual acuity (without glasses) were measured. For students with unaided visual acuity 0.3 or worse in either eye, subjective refraction was performed to obtain best-corrected visual acuity by trained optometrists. The cycloplegic procedure referred to the Anyang Childhood Eye Study [10]. Cycloplegia was achieved with two drops of 1% cyclopentolate and 1 drop of Mydrin P at a 5-min interval. Refractive status was measured using an autorefractor (KR-800, Topcon, Tokyo, Japan) before and after cycloplegia.

### Assessment of visual impairment

The prevalence of visual impairment and blindness were calculated using uncorrected (unaided), presenting, and best-corrected visual acuity [11]. Visual acuities evaluated via logMAR categories were defined as normal/near normal (≤ 0.2 in both eyes), unilateral visual impairment (0.2 in one eye only), mild visual impairment (0.3–0.5 in the better eye), moderate visual impairment (0.6–0.9 in the better eye), and blindness (≥ 1.0 in the better eyes). The causes of visual impairment of 0.3 or worse were assessed by the ophthalmologist.

### Definitions

Diagnosis was based on the following definition: (1) Refractive error was assigned routinely if visual acuity improved to ≤0.2 with refractive correction. (2) Myopia was defined as spherical equivalent refraction ≤ − 0.50 D, and (3) hyperopia as spherical equivalent refraction ≥ + 2.00 D. Children were considered myopic with at least one eye was diagnosed, and hyperopic with hyperopia at least one eye, so long as neither eye was myopic. Children were considered emmetropic if neither eye was myopic or hyperopic. (4) Unilateral amblyopia was considered as a 2-line interocular difference in eyes with BCVA 0.2 or worse. And at least one of the following risk factors must be presence: strabismus or history of strabismus surgery, past or present obstruction of visual axis, anisometropia consistent with the worse eye (≥1.00 D SER anisohyperopia, ≥3.00 D SER anisomyopia, or ≥ 1.50 D anisoastigmatism). Bilateral amblyopia was defined as bilateral BCVA >0.3. And there must be presence of bilateral visual axis obstruction or bilateral ametropia (≥4.00 D SE hyperopia, ≥6.00 D SE myopia, or ≥ 2.50 D astigmatism) [12]. Estimates of refractive error prevalence were based on children with successful cycloplegia in both eyes [11].

Presenting visual acuity was defined by the detected visual acuity when tested wearing currently available refractive correction, if any. Best-corrected visual acuity was the visual acuity achieved by subjects tested with pinhole or refraction. Corneal opacity was considered as the cause of vision loss in an eye if there was an easily visible opacity overlying the pupil to the extent that at least part of the pupil margin is blurred. Retinal disease was assigned the cause of vision loss in an eye found with chorioretinitis, optic disk atrophy, macular hole, and inherited retinal diseases. Cataract was assigned if a pupil appeared grey or white when examined with oblique light in a shaded or darkened area [13]. Children requiring medical or surgical treatment were recommended for eye clinic referral.

### Data management and analysis

Clinical examination forms were verified at least twice to evaluate integrity and precision before entered into the database using Epidata software 3.1 (The Epidata Association, Odense, Denmark) by two separated individuals.

Statistical analysis was performed using SPSS software (version 20.0). Means ± standard deviation, frequencies, and percentages were used to summarize the characteristics of the research subjects. The association between refractive status and the gender and ethnicity was explored using logistic regression. Confidence intervals and *P* values (significant at the *P*<0.05 level) for prevalence estimates and regression models were calculated and adjusted for clustering effects associated with the sampling design [9].

## Results

### Demographics of study population

A final of 1856 participants completed all the examinations, with an overall response rate of 95.52%. The mean age of students was 6.82 ± 0.47 years (median age, 6.78 years; range, 5.89 to 10.32), 984 (53.0%) were males, and 1762 (94.9%) were Tibetan minority nationalities.

Distribution of Visual Acuity and Prevalence of Visual Impairment.

Distribution of uncorrected visual acuity and best-corrected visual acuity is presented in Table 1. Measurements of uncorrected visual acuity were not possible in 2 children. By uncorrected visual acuity, normal/near normal visual acuity in at least one eye was found in 1627 children (87.8%). Two hundred twenty-seven children (12.2%) were visually impaired in both eyes, and 2 (0.1%) of them were blind in both eyes.

**Table 1:** Distribution of Uncorrected and Best-Corrected Visual Acuity

	Uncorrected Visual Acuity			Wearing glasses		Presenting Visual Acuity			Best corrected Visual Acuity		
Visual Acuity Category	n	%	95%CI	n	%	n	%	95%CI	n	%	95%CI
Normal/near normal	1450	78.2	76.33–80.09	6	0.3	1454	78.4	76.55–80.30	1735	93.6	92.47–94.70
Unilateral impairment	177	9.5	8.21–10.88	6	0.3	183	9.9	8.51–11.23	69	3.7	2.86–4.58
Mild impairment in better eye	205	11.1	9.63–12.48	3	0.2	198	10.7	9.27–12.90	46	2.5	1.77–3.19
Moderate impairment in better eye	20	1.1	0.61–1.55	1	0.1	17	0.9	0.48–1.35	3	0.2	0.00–0.34
Blind in both eyes	2	0.1	0.00–0.26	0	0	2	0.1	0.00–0.26	1	0.0	0.00–0.16
All	1854	100		16	0.9	1854	100		1854	100	

*CI* confidence interval

Normal/near normal means log MAR visual acuity ≤0.2 in both eyes

Unilateral visual impairment means log MAR visual acuity ≤0.2 in one eye only

Mild visual impairment means log MAR visual acuity 0.3–0.5 in the better eye

Moderate visual impairment means log MAR visual acuity 0.6–0.9 in the better eye

Blindness means log MAR visual acuity ≥1.0 in the better eye

For children wearing glasses in daily life, presenting visual acuity was recorded with their current refractive correction. Twelve out of 16 children achieved normal/near normal visual acuity in at least one eye. A 4.4% reduction of bilateral visually impairment was noticed over uncorrected vision without glasses.

After subjective refraction was performed to achieve best-corrected visual acuity, bilateral visual impairment was reduced to 2.7% (*n* = 50). And only 1 child remained bilateral blind. Nearly 80% (78.0%) children with bilateral visual impairment could reach normal/near-normal vision in at least one eye with refractive correction. These findings illustrate the potential benefit of spectacles in 77.0% of the children who had bilateral vision impairment.

### Refractive data

**Fig. 1:**
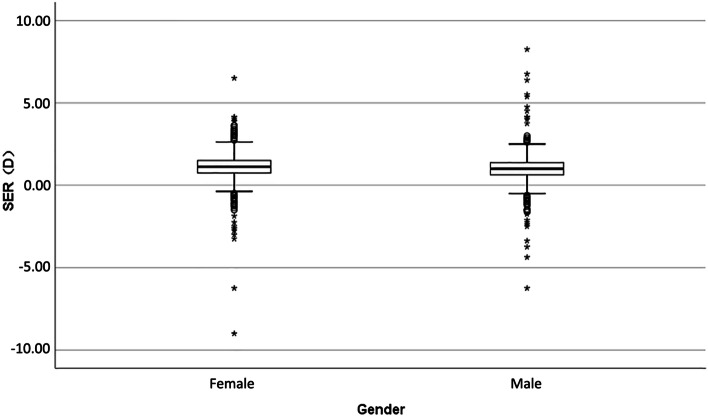
Distribution of spherical equivalent refractive error (D) in right eyes by gender

A total of 1837 (99.0%) children had cycloplegic autorefraction performed successfully on both eyes. Spherical equivalent in right eyes in boys (1.03 ± 0.90 D) was less positive compared to girls (1.12 ± 0.94 D), as shown in Fig. 1 (*P* = 0.025). The prevalence of myopia and hyperopia was 4.8 and 12.3% separately as shown in Table 2. Girls were more often hyperopic than boys.

**Table 2:** Prevalence of Refractive Status by Gender and Ethnicity

	Myopia			Hyperopia		
	n	%	95%CI	n	%	95%CI
Gender						
Boys	44	4.5	3.19–5.78	93	9.5	7–65-11.31
Girls	44	5.1	3.59–6.50	132	15.1*	12.76–17.52
Ethnicity						
Tibetan	82	4.7	3.68–5.65	211	12.0	10.48–13.51
Other	6	6.4	1.44–11.32	14	14.9	7.70–22.09
All	88	4.8		225	12.3	

*CI* confidence interval

*P<0.001

The prevalence of astigmatism was 44.8% in total as shown in Table 3. There was no statistically significant differences between each gender and ethnicity groups, either in low (cylinder value − 1.75D to − 0.75D) or high astigmatism (cylinder value ≤ − 2.00D).

**Table 3:** Prevalence of Astigmatism* by Gender and Ethnicity

Cylinder Value (D)	−1.75 to − 0.75		≤ − 2.00		All	
	n	%	n	%	n	%
Gender						
Boys	359	36.5	86	8.7	445	45.2
Girls	300	34.4	87	10.0	387	44.4
Ethnicity						
Tibetan	632	35.9	167	9.5	799	45.3
Other	27	28.7	6	6.4	33	35.1
All	659	35.5	173	9.3	832	44.8

Data are presented as number (%) of children in each gender or ethnicity groups

*Categorized using the cylinder value in the eye with greater astigmatism by cycloplegic refraction

### Risk factor analyses

Hyperopia was significantly associated with female gender (*P*<0.001), while there were no differences between gender and myopia or astigmatism. No significant association of ethnicity and myopia, hyperopia and astigmatism was observed.

### Ocular abnormalities

Tropia was present in 67 (3.6%) children. In all, 42 (62.7%) of them were exotropia. Exterior and anterior segment abnormalities were observed in 35 (1.9%) of the 1856 children: ptosis was observed in 3 eyes of 3 (0.2%) children; corneal abnormalities were present in 13 eyes of 11 (0.6%) children; pupillary abnormalities were noted in 3 eyes of 2 (0.1%) children; lenticular abnormalities were present in 15 eyes of 9 (0.5%) children; and other anterior segment abnormalities were observed in 12 eyes of 10 (0.5%) children.

### Causes of visual impairment

Visual impairment was mainly associated with refractive error, as shown in Table 4. A total of 285 children with uncorrected visual acuity 0.3 or worse in at least one eye attained visual acuity 0.2 or better in both eyes with refractive correction. A total of 83 (2.9%) children presented with best-corrected visual acuity 0.3 or worse in right eye. Among them, 33 were unilateral amblyopia (*n* = 10) or bilateral amblyopia (*n* = 23), 7 with a tropia; 2 with anisometropia 2.00 spherical equivalent diopters or more. Other causes of visual impairment were uncommon.

**Table 4:** Causes of Uncorrected Visual Acuity (UCVA) of 0.3 or Worse

Cause	Eyes with UCVA 0.3 or Worse		Percent Prevalence in the Population
	n	%	
Refractive error	285	70.5	15.4
Amblyopia	33	8.2	1.8
Corneal opacity	2	0.5	0.1
Cataract/posterior capsular opacification	1	0.2	0.1
Other	3	0.7	0.2
Unexplained cause	54	13.4	2.9

## Discussion

Various visual impairment disorders, such as refractive error, are related to several impact factors, and there are significant differences between regions, living environments, medical care and nationalities [14–16]. Because of the geographical remoteness from inland and backward economy, little information is available on the prevalence of ocular diseases in Tibetan children. The Lhasa Childhood Eye Study provides evaluations of the prevalence of refractive error and visual impairment situations in grade one children in Lhasa.

The current Lhasa Childhood Eye Study reported a 21.6% of the research population presenting with visual acuity of 0.3 or worse in at least one eye in daily living condition, which decreased to 6.4% with best corrected vision. With presenting visual acuity, 11.7% remained bilateral visual impaired (worse than 0.3 in better-seeing eye), which is significantly higher than the Ireland Eye Study (6–7 years 3.43%) [17], and the Northern Ireland Childhood Errors of Refraction study (6–7 years 1.5%) [18]. The higher percentage of poor presenting visual acuity may result from the poor economy and shortage of necessary glasses. A total of 177(78.0%) out of the 227 children with bilateral visual impairment could achieve normal/near-normal vision in at least one eye with refractive correction. Only 10 of them achieved normal/near normal visual acuity in at least one eye with their own glasses. A total of 33 out of 83 children with best-corrected visual acuity 0.3 or worse in right eye had asthenopia.

The major cause of visual impairment, such as refractive error and amblyopia, are largely preventable or treatable in early age [19, 20]. More attention should be paid to such a high prevalence of visual impairment of children in a remote low-incoming area. School-based screening programs provide the platform for inspecting a scalable number of children. School-age children comprise about a quarter of the population in developing countries and are easy to intervene. Besides, some conditions that can become permanent could be eliminated in early stage with a lower cost, such as refractive error and amblyopia. After proper examinations and identifying at risk children, more appropriate eye care services could be provided. To our knowledge, there is no cohort study following up the refractive status and visually impairment being implemented in Lhasa. The Lhasa Childhood Eye Study will provide further information in the next 5 year follow-up and is believed to contribute to relieve the visual abnormality of Tibetan school children.

The myopia prevalence in Lhasa Childhood Eye Study (6.82 ± 0.47 years 4.8%) was broadly in line with that reported in the Poland (7 years 4.0%)and Aston Eye Study (6–7 years 5.7%) [21, 22], higher than that reported in the UK Northern Ireland Childhood Errors of Refraction study (6–7 years 2.8%) [18], Australia (6 years 1.6%) [23], South Africa (7 years 2.5%) [24], but lower than urban China inland (7 years,7.7% by autorefraction) [25]. This difference may result from outdoor activity duration and study burden between different countries and regions.

The Lhasa Childhood Eye Study hyperopia prevalence (6.82 ± 0.47 years 12.3%)was comparable with Australia (6 years 13.2%) [23], and non-white ethnic groups of the Ireland Eye Study (6–7 years 11.1%) [17], and considerably lower than that reported in the other ethnic groups of the Ireland Eye Study (6–7 years 25.8–35.4%) [17], Northern Ireland (6–7 years 26%) [18], and higher than urban China inland (7 years, 4.0% by autorefraction) [25]. Same as myopia prevalence, outdoor activity duration and study burden may be the reason of the difference.

The astigmatism prevalence in Lhasa Childhood Eye Study (6.82 ± 0.47 years 44.8%) was similar to that found in Native American children (5–16 years 42%) [26], and significantly higher than Ireland Eye Study (6–7 years 19.2%) [17], Northern Ireland (6–7 years 24%) and Australia (6 years 7.6%) [18, 23].

Evidence from Refractive Error Study in Children demonstrated that differences in the prevalence of myopia were associated with ethnicity [5, 11, 15, 27–29]. In consist with Qian et al. (carried out in primary and secondary schools in the Tibet Naidong district) [7], a lower prevalence of myopia in Tibet than China inland was witnessed. However, there were no significant associations of ethnic or gender with myopia or astigmatism in Lhasa Childhood Eye Study. The prevalence of hyperopia in females was significantly higher than that in males in our survey. The difference might result from the different lifestyle, such as outdoor activity [14, 30, 31]. Not inconsistent with the finding in Naidong, no difference of the prevalence of hyperopia between ethnicity was recorded in Lhasa Childhood Eye Study. It is possible that environmental and lifestyle differences underlie the difference among studies, as well as the difference of study design [4, 32].

There are a few limitations in this study. The prevalence of blindness and low vision may be underestimated for children with low vision in both eyes may dropped out school and went to schools for the blind, which might have biased the analysis of the distribution of eye diseases. The reason for children ware glasses is not recorded in the present article. In addition, we did not record sociodemographic characteristics that might have been associated with refractive error, such as outdoor activity and family income.

## Conclusions

Our data revealed a lower prevalence of myopia compared to China inland, but a significantly higher prevalence of visual impairment compared to developed county [1, 33]. It is clear that vision screening in schools accompanied by health education is an important approach to alleviate the influence of visual impairment. Our survey demonstrated the unmet need for the necessarity of corrective spectacles. Effective strategies are needed to eliminate this easily treated cause of significant visual impairment.

## Data Availability

The datasets used and analysed during the current study available from the corresponding author on reasonable request.
